# Complete Genome Sequences of the *Carlavirus Sweet potato chlorotic fleck virus* from East Timor and Australia

**DOI:** 10.1128/genomeA.00414-16

**Published:** 2016-05-26

**Authors:** Solomon Maina, Owain R. Edwards, Luis de Almeida, Abel Ximenes, Roger A. C. Jones

**Affiliations:** a School of Plant Biology and Institute of Agriculture, Faculty of Science, University of Western Australia, Crawley, Western Australia, Australia; b Cooperative Research Centre for National Plant Biosecurity, Canberra, Australian Capital Territory, Australia; c CSIRO Entomology, Floreat Park, Western Australia, Australia; d Seeds of Life Project, Ministry of Agriculture and Fisheries, Dili, East Timor; e DNQB-Plant Quarantine, Presidente Nicolau Lobato International Airport, Dili, East Timor; f Department of Agriculture and Food Western Australia, South Perth, Western Australia, Australia

## Abstract

We present here the first complete genome sequences of *Sweet potato chlorotic fleck virus* (SPCFV) from sweet potato in Australia and East Timor, and we compare these with four complete SPCFV genomes from South Korea and one from Uganda. The Australian, East Timorese, South Korean, and Ugandan genomes differed considerably from each other.

## GENOME ANNOUNCEMENT

To examine possible connectivity between viruses infecting important crops in Australia and South-East Asia, we studied sweet potato viruses from East Timor and Australia. *Sweet potato chlorotic fleck virus* (SPCFV) is a single-stranded RNA virus belonging to the genus *Carlavirus*, family *Betaflexiviridae* (1). It has not been reported from East Timor, but it was found once in Australia, from where a single SPCFV coat protein sequence (AusCan) is available under accession number EF990647 (2). Currently, only four complete SPCFV genomes from South Korea and one from Uganda are available in GenBank (1, 3). Isolate AusCan and an SPCFV isolate from East Timor were sequenced and their complete genomes were obtained.

Fifteen East Timorese sweet potato samples blotted onto Fast Technology for Analysis of nucleic acids (FTA) cards (4) were sent to Australia. A plant infected with isolate AusCan was planted, and a scion from it graft-inoculated to indicator plant *Ipomoea setosa*. Total RNA was extracted from the FTA cards and an *I. setosa* leaf sample with virus symptoms using the ZR Plant RNA MiniPrep kit (Zymo Research) and treated with RNase-free DNase (Invitrogen) measured using Qubit (Invitrogen). RNA integrity was confirmed using RNA screen Tape (TapeStation 2200, Agilent Technologies). Libraries were prepared from total RNA using a TruSeqstranded Total RNA sample preparation kit with Ribozero-Plant (catalogue number RS-122-2401, Illumina). Final size and concentration of each library was verified using Qubit and D1000 ScreenTape (TapeStation 2200, Agilent Technologies). Sequencing was by HiSeq 2500 using a TruSeq SBS kit V4 (Illumina) with 151 cycles of paired-end reads. The reads were assembled and the genomes were annotated using CLC Genomics Workbench version 6.5 (CLC bio) and Geneious version 8.1.7 (Biomatters) (5).

Only one FTA card sample (Tm37) collected in May 2015 from the Dili district in East Timor contained SPCFV. It yielded 14,721,488 reads and, after trimming, 14,546,337 remained. *De novo* assembly generated 1,307 contigs and 21,003 reads mapped to the contig of interest with coverage of 397×. Sample AusCan yielded 6,260,728 reads and, after trimming, 5,405,928 remained. *De novo* assembly generated 832 contigs with 333,024 reads mapped to the contig of interest, giving coverage of 6,302×. Both Tm37 and AusCan sequences had the six intact open reading frames (ORFs) typical of carlaviruses (6). Pairwise nucleotide identity between Tm37 and AusCan was 72%. The closest match to Tm37 by BLAST was 87% to KP715159 from South Korea, and to AusCan it was 87% to KP115606, also from South Korea. Although the Tm37 and AusCan genomes only had 72.4% nucleotide identities, these are within the *Carlavirus* spp. demarcation of <72% identity (7). This high SPCFV genome sequence divergence provides no evidence of connectivity between East Timor and Australia. The same applies to connectivity between either of them and the South Korean and Ugandan genomes, which differed by at least 13%.

### Nucleotide sequence accession numbers.

The sequences were deposited in GenBank under accession numbers KU720565 (Tm37) and KU707475 (AusCan).
